# Human papillomavirus (HPV) types 16, 18, 31, 45 DNA loads and HPV-16 integration in persistent and transient infections in young women

**DOI:** 10.1186/1471-2334-10-326

**Published:** 2010-11-11

**Authors:** Agnihotram V Ramanakumar, Otelinda Goncalves, Harriet Richardson, Pierre Tellier, Alex Ferenczy, François Coutlée, Eduardo L Franco

**Affiliations:** 1 Division of Cancer Epidemiology, McGill University, Montreal, Quebec, Canada; 2 Laboratoire de Virologie Moléculaire, Centre de Recherche du Centre Hospitalier de l'Université de Montréal, Montreal, Quebec, Canada; 3 Département de Microbiologie et Immunologie, Université de Montréal, Montreal, Quebec, Canada; 4 Department of Family Medicine, McGill University, Montreal, Quebec, Canada; 5 Department of Pathology and Obstetrics & Gynecology, the Sir Mortimer B. Davis-Jewish General Hospital and McGill University, Montreal, Quebec, Canada

## Abstract

**Background:**

HPV burden is a predictor for high-grade cervical intraepithelial neoplasia and cancer. The natural history of HPV load in young women being recently exposed to HPV is described in this paper.

**Methods:**

A total of 636 female university students were followed for 2 years. Cervical specimens with HPV-16, -18, -31, or -45 DNA by consensus PCR were further evaluated with type-specific and β-globin real-time PCR assays. Proportional hazards regression was used to estimate hazard ratios (HR) of infection clearance. Generalized estimating equations assessed whether HPV loads was predictive of HPV infection at the subsequent visit.

**Results:**

HPV loads were consistently higher among women <25 years old, and those who had multiple sex partners, multiple HPV type infections and smokers. HPV-16 integration was encountered only in one sample. Infection clearance was faster among women at lower tertiles of HPV-16 (HR = 2.8, 95%CI: 1.0-8.1), HPV-18 (HR = 3.5, 95%CI: 1.1-11.2) or combined (HR = 2.4, 95%CI: 1.8-6.2) DNA loads. The relationship between HPV-16 and HPV-18 DNA loads and infection clearance followed a clear dose-response pattern, after adjusting for age and number of sexual partners. GEE Odds Ratios for HPV persistence of the middle and upper tertiles relative to the lower tertile were 2.7 and 3.0 for HPV-16 and 3.8 and 39.1 for HPV-18, respectively. There was no association between HPV-31 or -45 DNA loads and persistence.

**Conclusions:**

The association between HPV load and persistence is not uniform across high-risk genital genotypes. HPV-16 integration was only rarely demonstrated in young women.

## Background

Sexually active women are at risk for genital infection by human papillomavirus (HPV). Most genital HPV infections regress within two years and only a minority of women will develop persistent HPV infection that could eventually cause cervical intraepithelial neoplasia (CIN). High-grade CIN (grades 2 and 3) are precursors of invasive cervical cancer. Although measuring persistence has prognostic value in understanding the natural history of HPV infection and CIN, there is a need for studying additional virological endpoints to assist in risk prediction.

HPV-16 DNA load seems to be independently associated with high-grade CIN and invasive cancer [1-6]. Most studies also reported that HPV load was an ancillary marker for persistent HPV infection [3,7-11]. HPV-16 or 18 infections are cleared more slowly than infections caused by other high-risk types [12]. Since the biological behavior of HPV types differ, the predictive value for persistence of HPV DNA load may also vary between types [13]. We know little about type-specific viral loads and their relation with clearance of HPV infection. Moreover, most studies on HPV viral load have focused on older women at risk for CIN. The evaluation of HPV viral load in recently-infected younger women remains largely unexplored.

Integration of high oncogenic risk HPV types (HR-HPV) is considered to be a key event in the progression of CIN to invasive cancer [14]. Recent data casts doubt on the notion that viral integration into the host genome is a marker of progression to CIN2/3 [15-20]. Indeed, integrated HPV-16 DNA can be detected in women with CIN-1 or normal cervical samples, although these results were not confirmed by others [21]. It is important to establish whether HPV integration occurs early in the course of HPV infection to assess its contribution to carcinogenesis. Overall, the longitudinal assessment of HR-HPV load and integration in the natural history of HPV infection considering various viral outcomes such as clearance and persistence has received little attention up to now. In 1996, we began a prospective cohort study of young women attending college in Montreal, Canada, to investigate the epidemiologic determinants of persistent and transient cervical HPV infections [22,23].

The focus of the current study was to assess prospectively, in this cohort of young women, the time course and association between HPV-16 integration, HPV-16, 18, 31 and 45 DNA loads, and type-specific viral outcomes.

## Methods

### Study subjects

Female students attending either McGill University or Concordia University Health Clinics were invited to participate if they intended to remain in Montreal for the next 2 years and had not been treated for cervical disease in the last 12 months [22]. A total of 636 female university students were recruited between November 1996 and January 1999, and were followed for 2 years; with clinic visits every 6 months. Detailed information was obtained at enrolment via a self-administrated questionnaire and changes in lifestyle characteristics were obtained at each follow-up visit with an abridged questionnaire, as described previously [22]. Two cervical samples were collected with an Accelon cervical biosampler at every visit. A Papanicolaou smear was prepared with the first sampler. The remaining cells along with those collected with the second sampler were processed for HPV testing. Informed consent was obtained from all study participants. The study was reviewed and approved by the Ethics committees from each participating institution.

### HPV DNA testing

HPV DNA testing has been described elsewhere [22]. Briefly, five μl of sample DNA purified with QIAamp columns (Qiagen, CA) were first amplified for β-globin with PC04/GH20 primers to demonstrate the integrity of extracted DNA. β-globin-positive specimens were further tested with the L1 consensus HPV primers MY09/MY11 and HMB01 using *AmpliTaq *gold (Roche Diagnostic Systems, Laval, Canada) and with the Line blot assay for the detection of 27 genital HPV genotypes [22].

### HPV-16, 18, 31 and 45 viral loads

A total of 382 specimens collected from 183 participants were positive for HPV-16, -18, -31 or -45 DNA. Nineteen women were infected concurrently with two or more of these genotypes. Extracted DNA from these samples was first screened for the presence of PCR inhibitors by amplification of internal controls for HPV-16, HPV-18, HPV-31 or HPV-45, and for ί-globin DNA, as described previously [18,24]. The presence of PCR inhibitors was suspected when 1000 copies of at least one internal control generated a signal corresponding to less than 700 copies, as previously described [25]. All samples were free of inhibitors. Two μl of processed sample were tested in duplicate for quantification of ί-globin DNA to estimate the cell content of samples [18,24]. HPV-16 E6 and HPV-18 E7 DNA was quantified using a standard protocol [26]. HPV-31 L1 DNA was measured with the assay described by Weissenborn et al. [27]. HPV-45 E6 DNA was amplified in a Light Cycler PCR and detection system (Roche Molecular Systems) in a 20-μl reaction mixture containing 1× DNA Master Hybridization Probe Mix with the Fast Start *Taq *DNA polymerase (Roche Molecular Biochemicals), 0.3 pmoles of each HPV-45 primer 45 E6-F (nucleotide position 463-486; 5'-TTAAGGACAAACGAAGATTTCACA-3') and 45 E6-R (nucleotide position 670-647; 5'-ACACAACAGGTCAACAGGATCTAA-3'), and 50 nM of fluorogenic 45 E6-TM probe (nucleotide position 491-514; FAM-5'-AGCTGGACAGTACCGAGGGCAGTG-3'-TAMRA). Cycling parameters included an activation step at 95°C for 10 min followed by 50 cycles at 95°C for 15 sec, 60°C for 5 sec and 65°C for 45 sec. For each of the four genotypes analyzed, cycle thresholds obtained for each sample were compared to those of a titration curve obtained by serial ten-fold dilutions of HPV-16, 18, 31 or 45 plasmids in a fixed amount of 75 ng of human genomic DNA (Roche Diagnostics) in 10 mM Tris-HCl [pH 8.2]. Each assay consistently detected 10 HPV DNA copies (data not shown). HPV viral loads were expressed as the number of HPV DNA copies per cell.

### HPV-16 integration assays

The presence of integrated HPV-16 was investigated with real-time PCR assays targeting E6 and E2, as previously described [28]. Since disruption of the E2 gene often results in HPV integration, detection of a greater quantity of HPV-16 E6 compared to HPV-16 E2 strongly suggests the presence of integrated HPV-16 DNA [16,17]. Two μl of each processed sample was tested in duplicate in each HPV-16 E6 and E2 assays. Cycle thresholds were compared to those of serial ten-fold dilutions of an HPV-16 plasmid in a fixed amount of 2,000 copies of human genomic DNA (Roche Diagnostics). We assumed that HPV-16 DNA was integrated into the host genome if the ratio of copy numbers for HPV-16 E6 and E2 (HPV-16 E6/E2) in a specimen was two or higher [18,28,29].

HPV-16 integration was confirmed by restriction site PCR (RS-PCR), a sequencing technique that demonstrates the presence of HPV-16 and human DNA junctions in the same amplicon [30]. Briefly, 9 HPV-16-specific primers spanning the entire HPV-16 genome were combined separately with 6 restriction site oligonucleotides designed to anneal on selected restriction sites on the human genome, in a two-step hemi-nested PCR performed in a 9600 Thermal Cycler. Amplicons were migrated in a 2% agarose gel stained with ethidium bromide. When visible bands were obtained, direct double-stranded PCR-sequencing was done by a cycle-sequencing method (BigDye terminator ready reaction kit, Perkin-Elmer) using the internal primers and a sequencing primer on 20 ng of purified amplicon.

### Statistical Methods

We used Wilcoxon rank sum tests to compare type-specific viral loads among visits. The strength of the correlations among log-transformed viral load measurements at entry and follow-up visits for all four HPV types was measured using Pearson correlation coefficient (r). Geometric means of viral loads were calculated as a function of selected characteristics reported at the index visit of first positivity for a specific HPV type. The Kaplan-Meier technique was used to estimate the cumulative probability of infection clearance as a function of time since enrolment into the study. Proportional hazards regression was used to estimate the relative rate of clearance of type-specific infection, stratified by tertiles of the respective viral load distributions [31]. Logistic generalized estimating equations (GEE) were used to assess whether viral load measured at a given visit was a predictor of persistent HPV infection at the subsequent visit [32,33]. Crude and adjusted (for age and sexual partners) odds ratios (OR) between persistent HPV at visit (t) and viral load at visit (t-1) within specified periods of follow-up were calculated to measure the magnitude of the longitudinal associations.

## Results

The 636 women enrolled in the McGill-Concordia cohort contributed 2694 completed visits (mean of 4.2 visits/subject) during an average of 21.5 months of follow-up. Nearly all cervical specimens (n = 2,626, 97.4%) were suitable for HPV testing. Among those enrolled in the cohort, 68% completed all 5 visits, contributing a total of 13,353 woman-months of follow-up. The mean age of participants was 23 years (median, 21 years; range, 17-42 years). Four in five women reported that they were Caucasian, and one in four are smokers. Nearly half of participants had more than 4 lifetime sexual partners. The prevalence (any HPV = 29%, high risk HPV = 22%), cumulative incidence rates (cumulative incidence of any HPV = 36% and high risk HPV = 29% in 2 years) and mean duration of HPV infections have been reported elsewhere [22]. High-grade and low-grade squamous intraepithelial lesions (SIL) were found in only 4 and 49 Pap smears, respectively, which precluded the analysis of association between HPV loads and SIL.

**Figure 1:**
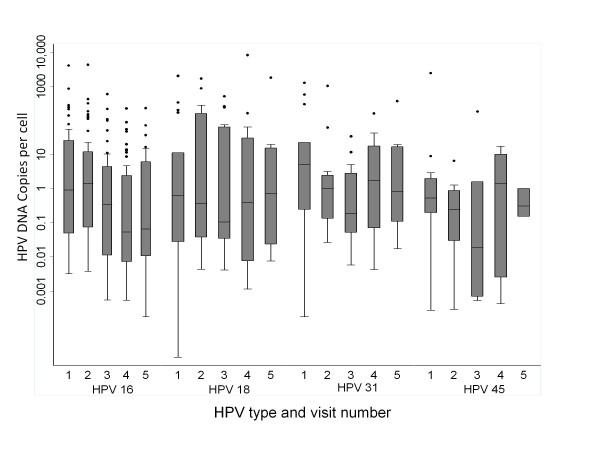
**HPV loads (HPV DNA copies per cell) at enrolment and at follow-up visits (total 5 visits) among HPV-positive women for the 4 genotypes studied (Y-axis in log scale)**. The length of each box corresponds to the interquartile range, with the top boundary of the box representing 75th and bottom boundary the 25th percentile. The horizontal line in the box indicates the median value. Outlier values are shown in circles outside the boxes.

Samples positive for HPV-16 (n = 220 from 104 women, mean of 2.1 samples/woman), HPV-18 (n = 80 from 43 women, mean of 1.9 samples/woman), HPV-31 (n = 75 from 36 women, mean of 2.1 samples/woman), or HPV-45 (n = 33 from 19 women, mean of 1.7 samples/woman), were further tested with type-specific quantitative real-time PCR assays. Nineteen samples contained more than one of the studied genotype. Descriptive statistics of log-transformed HPV loads stratified by baseline and follow-up time points are presented in Figure 1. Except for the fluctuation in HPV-45 loads, which could be due to the small number of samples, we observed no significant between-visit variation among type-specific viral loads.

**Figure 2:**
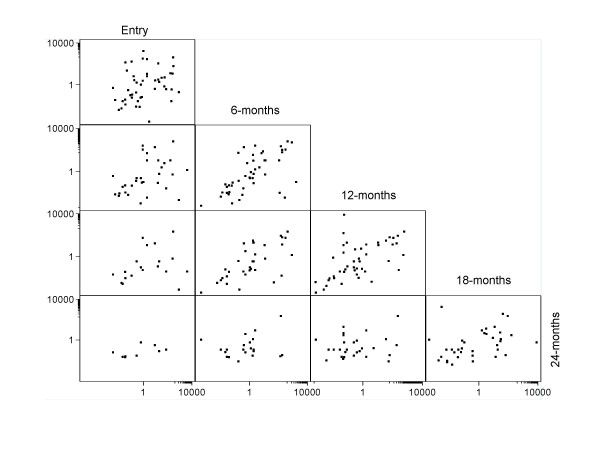
**Between-visit correlation graphs of viral load measurements of four combined HPV types (HPV-16, 18, 31, 45) at enrolment and follow-up visits (see material and methods)**. For example, the upper left graph shows viral load at entry versus viral load at 6 months, the lower left graph shows viral load at entry versus viral load at 24 months.

To investigate the consistency of type-specific viral load over time we calculated correlation coefficients for measurements taken in all possible pairs of visits (Table 1). HPV loads were significantly correlated only when considering consecutive visits. The strength of between-visit correlations seemed to become weaker as time progressed. Stronger correlations between consecutive visits were found in women with HPV-16 infection. Correlation coefficients of HPV loads were non-significant when visits 12 months apart were compared, except when viral loads for all four HPV types were combined (Table 1 and Figure 2).

**Table 1:** Between-visit correlation coefficients of HPV type-specific and combined viral load measurements at entry and follow-up visits in the McGill-Concordia cohort.

		Correlation coefficient with visit at			
		
HPV type	Visit at	6 months	12 months	18 months	24 months
16	Entry	0.5232* (30)	0.3181 (20)	0.2046 (14)	0.4583 (6)
	
	6 months		0.7561** (27)	0.6764** (21)	0.4282 (10)
	
	12 months			0.8295** (30)	0.5810* (17)
	
	18 months				0.8043** (24)

18	Entry	0.0103 (10)	0.5481 (5)	ND	ND
	
	6 months		0.3132 (11)	0.6612 (5)	0.2290 (4)
	
	12 months			0.0173 (8)	0.1732 (6)
	
	18 months				0.1655 (8)

31	Entry	0.9562** (6)	0.4242 (5)	0.5133 (5)	0.2410 (3)
	
	6 months		0.2427 (6)	0.8063* (5)	0.5138 (5)
	
	12 months			0.1930 (11)	0.3575 (7)
	
	18 months				0.7289* (12)

45	Entry	0.3648 (6)	ND	ND	ND
	
	6 months		0.7376 (3)	ND	ND
	
	12 months		ND	0.9745 (3)	ND
	
	18 months				ND

Above HPV types combined	Entry	0.4122* (53)	0.4044* (37)	0.3769* (23)	0.6103* (12)
	
	6 months		0.6190** (49)	0.6126** (36)	0.2280 (22)
	
	12 months			0.5752** (49)	0.1391 (33)
	
	18 Months				0.4116* (44)

HPV loads were measured with type-specific PCR assays and expressed as logarithmic transformation before computing correlations. Pearson correlation coefficients are shown with number of visit pairs jointly positive for the stated type or combination in parenthesis. ND: not determined because only 2 pairs were evaluable.

* denotes significance at 5% level of significance; ** denotes significance at 1% level of statistical significance.

Table 2 shows geometric mean viral loads and respective 95% confidence intervals according to selected characteristics determined at the first visit in which positivity was detected for each given HPV type. HPV DNA loads were slightly higher among women younger than 25 years of age, with >2 lifetime sex partners, who were regular oral contraceptive users, and with sequential HPV co-infections but not those with concurrent co-infections. However, confidence intervals were largely overlapping for most comparisons. HPV DNA loads did not show a consistent pattern of variation according to smoking categories or condom use.

**Table 2:** Means of HPV viral loads according to selected characteristics at the first occurrence of positivity for a given HPV type

	HPV 16			HPV 18			HPV 31			HPV 45		
	
Characteristic*	N	Mean	95% CI	N	Mean	95% CI	N	Mean	95% CI	N	Mean	95% CI
Age												
<25 years	72	0.47	0.19-1.19	32	0.93	0.18-4.96	31	2.07	0.54-7.97	14	0.26	0.03-2.62
25+ years	31	0.21	0.05-1.01	11	0.08	0.03-0.46	5	0.45	0.04-5.33	5	0.09	0.01-8.02

Coinfection, other HPVs												
None #	10	0.17	0.02-1.24	5	0.05	0.01-0.25	4	1.88	0.10-35.6	3	0.09	0.01-8.03
Sequential	56	0.56	0.18-1.70	21	1.61	0.14-18.18	17	1.29	0.12-14.1	8	0.06	0.00-3.37
Concurrent	38	0.25	0.06-0.93	17	0.62	0.05-7.09	15	1.76	0.29-10.9	8	0.41	0.00-18.58

Smoking												
Never	60	0.34	0.11-1.04	23	0.16	0.02-1.05	22	0.66	0.09-4.49	11	0.17	0.03-8.28
Current	25	0.79	0.21-2.91	15	1.29	0.18-9.36	8	1.80	0.38-8.56	3	0.32	0.05-2.46
Former	18	0.13	0.02-1.07	5	3.86	0.04-37.47	6	21.87	2.26-211.3	5	0.17	0.03-11.18

Condom use												
No	15	0.09	0.01-1.05	10	0.72	0.01-44.43	4	0.49	0.02-122.	3	0.03	0.003-2.48
Used sometimes	44	0.78	0.21-2.82	17	0.35	0.04-3.34	13	6.09	0.70-53.1	12	0.33	0.02-6.19
Regular use	39	0.51	0.17-1.48	15	0.72	0.08-6.92	16	1.01	0.15-6.68	5	0.22	0.02-16.23

Oral contraceptive use												
No	23	0.28	0.04-1.80	13	0.22	0.02-2.18	10	0.51	0.04-2.09	7	0.13	0.01-1.57
Infrequent	63	0.42	0.16-1.08	25	0.69	0.13-3.65	21	0.91	0.19-4.34	10	0.46	0.02-9.44
Regular	7	1.10	0.18-67.6	4	1.92	1.3-3.82	4	7.4	2.08-26.7	2	0.98	0.01-54.21

Sexual partners												
One	5	0.05	0.01-6.12	5	0.02	0.04-9.03	2	0.80	0.03-205.	5	0.06	0.01-3.60
2-3	24	0.72	0.16-3.16	7	2.30	0.04-146.1	13	3.32	0.31-34.9	2	0.06	0.005-6.57
4+	75	0.34	0.14-0.90	31	0.59	0.13-2.62	21	1.09	0.23-5.04	12	0.41	0.03-5.61

* Defined at the time of first occurrence of positivity for the stated HPV type.

# Concurrent coinfection defined as the detection of more than one HPV type in the cervical cell specimen collected at the first visit when the stated type was found. Sequential coinfection defined as additional HPV types detected at different visits than the one when the stated type was found.

**Figure 3:**
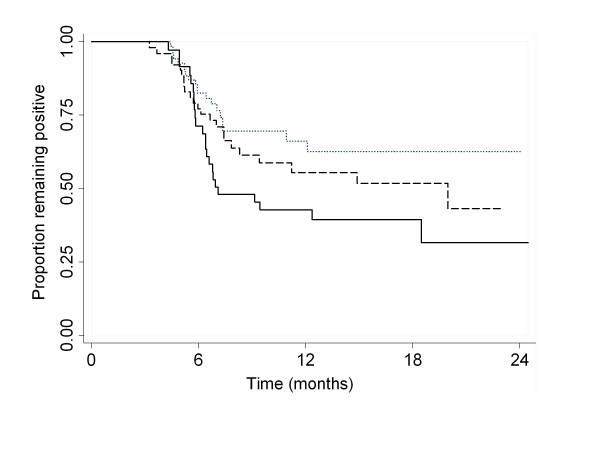
**Cumulative positivity rate for infections with HPV types 16, 18, 31, and 45 according to tertiles of cumulative viral load for these 4 types**. Unit of analysis is an individual infection episode, which makes observations not independent as a subject may contribute multiple episodes. Dotted line: upper tertile; dashed line: middle; solid line: lower tertile viral load.

Once detected, 30% of HPV-16, 44% of HPV-18, 63% of HPV-31 and 55% of HPV-45 infections cleared within 6 months. Table 3 shows the relative rates of clearance of HPV infection according to the HPV DNA load for each genotype classified into tertiles. For HPV-16 and HPV-18, infection clearance was inversely associated with viral load but no clear pattern of association emerged for HPV-31 and HPV-45. Owing to the preponderance of HPV-16 and HPV-18 in the cohort the overall association between combined viral loads and clearance maintained the inverse pattern seen with the latter types. Figure 3 shows the cumulative rates of positivity by tertile of the combined viral load distribution.

**Table 3:** Relative rates* of type-specific and combined HPV infection clearance according to viral load

			Relative rate (95%CI)	
			
HPV type	Viral load tertile	No. of Events/No. Eligible cases	Unadjusted	Adjusted
16	Third	5/27	1.0	1.0
	Second	8/27	1.52 (0.49-4.69)	1.31 (0.41-4.20)
	First	13/27	2.55 (0.90-7.24)	2.38 (0.82-6.89)

18	Third	4/13	1.0	1.0
	Second	3/13	1.64 (0.31-8.78)	1.14 (0.18-7.35)
	First	11/13	4.76 (1.23-18.45)	4.61 (1.17-18.05)

31	Third	6/10	1.0	1.0
	Second	3/10	0.87 (0.21-3.69)	0.80 (0.19-3.44)
	First	2/9	0.67 (0.16-2.75)	0.66 (0.15-2.97)

45	Third	3/6	1.0	1.0
	Second	4/6	1.99 (0.21-19.02)	1.59 (0.15-16.91)
	First	3/6	2.25 (0.20-25.09)	2.06 (0.17-24.27)

Above HPV types combined	Third	20/55	1.0	1.0
	Second	22/55	1.45 (0.79-2.64)	1.49 (0.81-2.74)
	First	24/56	2.11 (1.16-3.86)	2.21 (1.19-4.10)

* Using Cox proportional hazards regression. Rate of clearance for third viral load tertile (highest) taken as referent. For the analysis of combined types the observations are not independent and required a robust variance estimator. Adjusted relative rates were estimated in models that included age and sexual partners as covariates.

The association between HPV viral load and persistence of infection was investigated for each genotype by calculating crude and adjusted (for age and number of sexual partners) GEE ORs (Table 4). HPV persistence was significantly associated with intermediate and high viral load levels at a preceding visit for HPV-16 and HPV-18. There was no consistent effect of viral load on persistence for HPV-31 and HPV-45 infections.

**Table 4:** Odds ratios for associations between persistent HPV infection at visit (t) and viral load at visit (t-1) estimated via GEE models

			OR and 95% CI	
			
HPV type	Viral load tertile	No. of Events/No. Eligible cases	Crude	Adjusted*
16	First	29/54	1.0	1.0
	
	Second	42/54	2.97 (1.25-7.03)	2.88 (1.22-6.87)
	
	Third	40/54	2.47 (1.15-5.32)	2.45 (1.14-5.26)

18	First	7/21	1.0	1.0
	
	Second	13/21	5.24 (0.65-42.52)	4.33 (0.80-23.61)
	
	Third	17/21	10.23 (1.39-75.41)	11.33 (1.90-67.59)

31	First	14/18	1.0	1.0
	
	Second	13/18	0.73 (0.13-4.00)	0.77 (0.15-3.80)
	
	Third	8/18	0.22 (0.04-1.28)	0.24 (0.05-1.28)

45	First	5/9	1.0	1.0
	
	Second	4/10	0.66 (0.05-7.99)	0.46 (0.04-5.02)
	
	Third	5/8	1.97 (0.12-32.16)	0.92 (0.06-14.02)

Events are considered as HPV infections with the stated type at visits at times t = 2, 3, 4, and 5 as a function of viral load determined at the preceding visit.

*Adjusted for age and number of lifetime sexual partners

We then investigated if persistent HPV-16 infection and/or high HPV-16 viral loads in young women could result in integration of HPV-16 DNA into the human genome. Forty-eight specimens were excluded from this analysis because they contained <15 copies of HPV-16 DNA per μl of extracted DNA. HPV-16 E6 and E2 DNA were thus quantitated on the remaining 172 HPV-16-positive samples that had sufficiently high viral load to permit reliable measurement of integration. The mean HPV-16 E6 viral load on the selected samples was 57.5 ± 324.6 DNA copies per cell (95% confidence interval, 8.6-106.4; median, 0.92, range 0.0001-4084.7 copies per cell). The mean HPV-16 E6/E2 ratio was 0.97 ± 0.25 (95% confidence interval, 0.93-1.01; median, 0.97; range, 0.5-2.48). Of the 172 specimens analyzed, 169 had a HPV-16 E6/E2 ratio <1.5, two samples had HPV-16 a E6/E2 ratio >1.5 and <2.0 (1.54 and 1.70) and one had a ratio above 2. Samples collected at consecutive visits from the two participants with HPV-16 ratios of 1.54 and 1.70 yielded HPV-16 E6/E2 ratios near or below 1.0 (data not shown). The only sample collected from a participant that generated a HPV-16 E6/E2 above 2 (ratio of 2.48) contained 0.94 copy/cell of HPV-16 E6 DNA and was obtained at the last visit. HPV-16 was detected only at the last of five visits attended by this participant. Normal cytology smears were obtained at the first four visits for this participant while a low-grade SIL smear was obtained at the fifth visit. RS-PCR was performed on the 18 samples that generated a HPV-16 E6/E2 ratio ≥1.2. Despite using several primer combinations, we could not demonstrate the presence of cellular and HPV junctions in any of the samples tested. A minimal amount of 35 μg of cellular DNA per test was analyzed for the only sample with a ratio >2, unsuccessfully, which could have limited our ability to sequence HPV-human DNA junctions.

Since we detected HPV-16 integrated forms only once, we investigated if quantity of cellular DNA introduced in the quantitative assays hampered our ability to measure HPV-16 E6 and E2, as reported by another group [34]. When mixtures of episomal HPV-16 and DNA extracted from SiHa cells were tested, we observed interference with quantitation of HPV-16 E2 and E6 with DNA extracted from 10^6 ^and from 10^4 ^SiHa cells, respectively (data not shown). One thousand copies of episomal HPV-16 DNA was detected without loss of signal when tested in a mixture of DNA extracted from up to 200,000 cells in the HPV-16 E2 assay and up to 40,000 cells in the HPV-16 E6 test (Table 5). Interference of HPV-16 quantitation by input DNA was not an issue in our study since all samples tested contained ≤39,200 copies of cellular DNA per test.

**Table 5:** Interference of background human DNA in quantitation of HPV-16 DNA with HPV-16 E6 and HPV-16 E2 real-time PCR assays.

No. of copies of human DNA	Quantitation of 1000 copies of episomal HPV-16 DNA with real-time PCR assays	
	**HPV-16 E2**	**HPV-16 E6**

0	933 ± 13	1150 ± 39

8 × 10^3^	1080 ± 35	1114 ± 7

1 × 10^4^	1097 ± 38	1179 ± 148

2 × 10^4^	1026 ± 149	1109 ± 59

4 × 10^4^	1125 ± 15	1015 ± 46

6 × 10^4^	1173 ± 10	820 ± 30

8 × 10^4^	1151 ± 100	726 ± 19

1 × 10^5^	1082 ± 82	787 ± 32

2 × 10^5^	1071 ± 38	562 ± 20

1000 copies of HPV-16 DNA were amplified in a background of various copies of human DNA and detected in real-time PCR assays for quantitation of HPV-16 E6 and HPV-16 E2. Results are means ± 1 standard deviation of duplicate values. Repeat experiments gave similar results.

## Discussion

We measured HPV DNA load for four high-risk types (16, 18, 31, 45) with real-time PCR on a set of samples collected prospectively in young women. These four genotypes are amongst the most frequently detected in cervical cancer. Contrary to cross-sectional studies of older women, the four HPV genotypes were detected at similar loads and were not substantially different between women with single and multiple type infections [1,11,35].

The quantitative real-time PCR assays we utilized to estimate HPV loads were specific and reproducible [25,28]. The number of HPV DNA copies was normalized for cell content by quantitation of β-globin DNA. The HPV-16 integration assay was devised considering the genetic diversity of HPV-16 [28,36]. Using type-specific quantitative assays allowed isolating the effect of HPV type loads in multiple type infections. The cohort design also allowed testing longitudinal correlations between pairs of visits. Consistent measurements for HPV types 16, 18, and 31 were shown for the five visits with evidence of correlation between loads among visits within subjects, whenever statistical precision was sufficiently high.

As reported by others, younger woman (<25 years) harbored higher HPV loads than those >25 years of age [1]. These women were possibly exposed to HPV while they were immunologically naïve to HPV. We also observed a somewhat greater HPV burden among current and former smokers, although not consistently for all four HPV types. This finding could be explained by a possible defective cell-mediated immunity against HPV induced by tobacco [37]. Results from this cohort as well as those of others suggest that tobacco smoking may increase the duration of high-risk HPV infection [23,38]. This could be explained in part by the increased replication of HPV in women exposed to tobacco.

Although the regular use of condoms protects against most sexually transmitted infections, they are not as efficient against HPV infection [39]. We found a trend with the consistent use of condoms for having higher HPV loads for most genotypes. These results could be biased because use of condoms may be associated with risky sexual behavior or exposure to new sexual partners [23]. Condom use has been associated in one study with regression of CIN and clearance of HPV [40]. Although oral contraceptive use did not modify the duration of high-risk HPV infection in our cohort [23], HPV-18 DNA loads were markedly increased in women on oral contraceptives. Oral contraceptives may also be a proxy for a higher number of sexual partners.

HPV-16 and -18 loads were good predictors of the duration of infection with these types but the same finding was not held for HPV-31 and -45. HPV-16 load has been reported by others to be a stronger predictor for persistence or lesions than HPV-18, 31 or 33 loads [13]. There was a clear dose-response relationship between HPV load and persistence of HPV-16 and HPV-18 infections. We found that clearance rates depended largely on the level of HPV load. Viral-host interactions play an influential role in the clearance of viral infections [41]. HPV has developed several mechanisms to evade the host immune system [42]. Functional differences between HPV-encoded proteins could also explain why some types and variants have a better viral fitness with a greater ability to persist [14,41,43]. For HPVs 16 and 18, viral loads were greater with HPV co-infections at different visits (sequential) than concurrent co-infection. In recent studies, two or more oncogenic HPV types diagnosed concurrently did not confer an additional risk of developing lesions [44,45]. All but one study confirmed that sustained or increased viral loads, especially with HPV-16, were predictive of persistent infection [3,5,8,11]. In a cohort of nearly 6,000 women in France, women with HPV loads above 10 pg/ml were less likely to clear the infection, irrespective of the age of participants [8]. Similarly, another cohort study conducted in the Netherlands reported that women with low HPV-16 loads were five times more likely to clear HPV-16 infection [5]. In a third study conducted in Brazil, there was a dose-response relationship between increasing viral loads and risk of incident abnormal smear over time [3].

HPV-16 integration often results in the disruption of the E2 gene, leading to uncontrolled expression of HPV-16 oncoproteins [14,17]. HPV-16 integration could thus occur in the course of persistent HPV-16 infection and increase the likelihood of progression to higher grade lesions. Integration of the HPV-16 genome was believed to occur at the CIN-2,3 stage and beyond [14]. Recent studies support that integration can occur even in the absence of CIN [15,28]. In one study conducted in women age 15 to 19 years old [46], disruption of the E2 gene was demonstrated in up to 25% of incident HPV-16 infections, suggesting that HPV-16 E2 disruption was a common event occurring early during infection. In contrast to this latter study, HPV-16 E2 disruption was rare in our study population, perhaps because unlike Collins et al [46] who studied the entire E2 gene for disruption our assay only focused on the hinge region of E2. While disruption of E2 is thought to occur more frequently in the very early phases of infection in younger women at least one other study of older women followed longitudinally, observed approximately 50% of women with persistent or transient infections to have mixed integrated and episomal forms [11]. However, unlike our study, over 50% of these participants had Pap smear abnormalities. Furthermore, HPV integration was not found to be associated with persistence [11]. Results from other cohorts are needed to assess the rate of integration and to clarify the role of HPV integration on persistence at early stages of infection.

There are some limitations to our study. We cannot exclude the possibility that we may have underestimated HPV-16 integration due to HPV-16 disruption during integration in areas outside of the E2 hinge region. Nevertheless, HPV-16 E2 hinge is the most frequently disrupted site in studies conducted in North America [16].

The analytical sensitivity of real-time PCR assays for quantitation of HPV-16 E6 and E2 DNA utilized in this study has been demonstrated previously and shown to be excellent [18,28,26]. These reagents were optimized to avoid any influences of viral polymorphism on the efficiency of HPV-16 DNA amplification [28]. Nevertheless, the clinical sensitivity and specificity of these assays to detect HPV integration have not been thoroughly assessed. Reconstitution experiments have demonstrated that HPV-16 integration is detected with real time PCR assays measuring E6 and E2 when integrated HPV-16 forms are in 100-fold excess of episomal HPV-16 DNA [47]. Theoretically, a HPV-16 E6/E2 ratio above 1.0 could suggest integration. However, previous work on HPV-6, HPV-16 and -33 have demonstrated that HPV E6/E2 ratios below 2.0 results from assay variability rather than true differences between E6 and E2 quantities [2,18,29,34]. Real time PCR tests may thus be falsely negative for HPV-16 integration in the presence of low quantities of integrated forms and high quantities of episomal forms.

We could not confirm the presence of HPV-16 integration with a standard technique identifying the presence of HPV-human DNA junctions in the only sample with a HPV-16 E6/E2 ratio above 2.0. However, the quantity of sample that could be analyzed was limited. A recent report using a similar technique to demonstrate the presence of HPV-human junctions did not find HPV-16 integration in specimens from women with low-grade SIL [21]. Real-time PCR assays may be helpful to detect integrated HPV forms but further studies on greater number of specimens comparing different techniques for detection of integrated HPV-16 need to be conducted.

In our study, few women had abnormal smears, reducing our power to test the associations between HPV load and lesion outcomes. The time interval between visits can influence the assessment of persistence and clearance. The majority of women in our cohort returned within 6 months of each visit and there was only a small proportion of women whose time interval between visits extended beyond one year [22]. The association between higher viral loads and persistence would only be distorted if it had been associated differentially with time between visits. Given that the participants were unaware of their HPV and viral load status, this is an unlikely scenario. It is also unlikely that their behaviour and other risk factors will change in this short span of time. The same associations between HPV loads and persistence were obtained when HPV persistence was defined more stringently by using three consecutive HPV-positive visits for the same type.

Consecutive detection of HPV DNA is due to either ongoing viral replication, reactivation of latent infections or new infections [48,49]. The design of our study cannot discriminate between these three possibilities. Participants considered as having persistent infection could have indeed, been reinfected with another isolate of the same HPV type. However, this seems unlikely because women with persistent infection were all infected with the same intratypic HPV variant. Both prevalent and incident HPV infections were included in our analysis of persistence, increasing the power of our analyses. Although it may not have a direct implication on HPV clearance, the exact duration of prevalent cases is unknown. By including them we could have introduced a bias because a greater proportion of prevalent HPV infections represent persistent infections compared to incident infections. However, our conclusions did not change when we restricted our analyses to incident infections (data not shown). We also analyzed the entire cohort to utilize the clustered binary outcome of persistence, by using the 'visit number' as panel variable in logistic GEE models. Apart from the gain in precision when using multiple visits from the same women, the findings from these models were in agreement with those from Cox models that analyzed clearance in individual women by tertile of viral load. Therefore, we conclude that the bias incurred due to inclusion of prevalent cases is minimal. We also doubt that misclassification of HPV status may have affected our results since there were very few women with persistent type-specific infections who had an intervening visit with a negative HPV test result and more than 80% of the same type persistent infections occurred during consecutive visits [22].

## Conclusion

In conclusion, this study demonstrates a clear association between HPV load and persistence of HPV-16 and 18 infections in young women at the early stages of their sexual life. The association between viral load and infection clearance seems to be type specific and, except for age, it is not substantially affected by sociodemographic characteristics and other risk factors for infection. More longitudinal studies are needed to clarify the onset of HPV integration and its relationship with disease progression.

## Competing interests

Financial competing interests: None

Non-Financial competing interests: None

The authors declare that they have no competing interests

## Authors' contributions

AVR performed the statistical analysis and drafted the first version of the manuscript. OG performed viral load measurements and integration studies, and interpreted their findings. HR participated in the design and coordination of the study and collection of epidemiologic data. PT participated in the design of the study, oversaw recruitment of subjects, and collected samples. AF participated in the design of the study, read Pap smears, and provided expert oversight. FC helped design the study, supervised HPV genotyping, viral load, and integration assays, participated in the analysis of results and helped interpreting the results and drafting of the manuscript. EF was the study's principal investigator, and participated in its design and coordination, supervision of analyses, and drafting. All authors read and approved the final manuscript and subsequent revisions.

## Pre-publication history

The pre-publication history for this paper can be accessed here:

http://www.biomedcentral.com/1471-2334/10/326/prepub
